# Pediatric Ileocecal Burkitt Lymphoma Presenting as a Diagnostic Challenge After a Nondiagnostic Surgical Biopsy: A Case Report

**DOI:** 10.7759/cureus.103380

**Published:** 2026-02-10

**Authors:** Eyad S Alhudaithi, Khalid Asiri, Abdulmajeed Saad Mohammed Alshahrani, Ijeoma A Okwudire-Ejeh, Mohammed Alzahimah, Badriah G Alasmari

**Affiliations:** 1 Pediatric Gastroenterology, King Faisal Medical City for Southern Regions, Abha, SAU; 2 Pediatric Gastroenterology, Armed Forces Hospital Southern Region, Khamis Mushait, SAU; 3 Pediatric Gastroenterology, Khamis Mushait Maternity and Children Hospital, Aseer Health Cluster, Ministry of Health, Abha, SAU; 4 Histopathology, Khamis Mushait Maternity and Children Hospital, Aseer Health Cluster, Ministry of Health, Abha, SAU; 5 Pediatrics, King Khalid University Hospital, Ministry of Health, Abha, SAU; 6 Pediatrics, Armed Forces Hospital Southern Region, Khamis Mushait, SAU

**Keywords:** burkitt lymphoma, case report, extranodal lymphoma, nondiagnostic biopsy, pediatric gastrointestinal lymphoma, pediatric oncology

## Abstract

Burkitt lymphoma (BL) is a highly aggressive form of non-Hodgkin lymphoma that frequently affects children and often presents with nonspecific GI symptoms, making early diagnosis challenging. GI involvement is common and may closely mimic more prevalent pediatric conditions such as appendicitis or inflammatory bowel disease (IBD), leading to diagnostic delays. We report the case of a nine-year-old boy who presented with a five-month history of chronic abdominal pain, diarrhea, and unintentional weight loss. He was initially managed for suspected appendicitis, and an appendectomy was performed. During surgery, an incidental cecal mass was identified and biopsied; however, histopathological examination revealed only minimal histiocytosis, with no evidence of malignancy. Despite surgical intervention, the patient’s symptoms persisted. One month later, due to ongoing clinical concerns and to rule out IBD, a repeat endoscopic evaluation was undertaken, which demonstrated a large, ulcerated cecal mass. Histopathological analysis of repeat biopsies confirmed a diagnosis of high-grade B-cell lymphoma consistent with BL. Laboratory investigations at presentation were notable for marked leukocytosis, elevated erythrocyte sedimentation rate, and significantly increased lactate dehydrogenase levels, reflecting a high tumor burden. The current case report shows the diagnostic challenges of GI BL in children, underscores the risk of false-negative initial biopsies, and emphasizes the importance of maintaining a high index of suspicion in patients with persistent GI symptoms despite prior intervention.

## Introduction

Burkitt lymphoma (BL) is a highly aggressive, rapidly proliferating B-cell non-Hodgkin lymphoma and represents one of the most common pediatric malignancies worldwide [1]. The sporadic form, which predominates in North America and Europe, frequently involves the GI tract, with the terminal ileum being the most commonly affected site due to the high concentration of Peyer’s patches [2]. The clinical presentation of abdominal BL is often nonspecific and may include abdominal pain, distension, nausea, vomiting, and altered bowel habits. Such features contribute to a broad differential diagnosis, and BL may closely mimic common pediatric surgical emergencies, including acute appendicitis and intussusception [3,4].

Misdiagnosis remains a significant clinical challenge. Multiple case reports have described BL presenting as acute appendicitis, with the diagnosis often made incidentally following appendectomy through histopathological examination of surgical specimens [4-7]. Moreover, in other cases, the clinical, radiological, and endoscopic findings of GI lymphoma may closely resemble those of inflammatory bowel disease (IBD), particularly Crohn’s disease, with overlapping features such as ulceration, transmural involvement, inflammatory mass formation, and luminal strictures [8-10].

This case presents as an interesting diagnostic challenge in a nine-year-old boy with ileocecal BL. Despite persistent GI symptoms, the initial appendectomy and surgical biopsy were nondiagnostic, resulting in a delay in identifying the underlying malignancy. The definitive diagnosis was established only after repeat evaluation with endoscopy and targeted biopsy. This report highlights important diagnostic pitfalls and underscores the need for a high index of suspicion and prompt reevaluation when pediatric patients fail to improve as expected following initial management.

## Case presentation

According to his parents, a nine-year-old boy was electively admitted for diagnostic upper and lower GI endoscopy. They reported a five-month history of chronic right lower quadrant abdominal pain, persistent moderate diarrhea, progressive unintentional weight loss of approximately 5 kg, and chronic iron deficiency anemia. There was no history of fever, GI bleeding, night sweats, or significant family history of IBD or malignancy. His past medical history was unremarkable. His past surgical history was notable for a previous appendectomy performed approximately one year prior to this admission, with no reported postoperative complications. He was not receiving any regular medications and had no known drug allergies.

On admission, the patient appeared pale and underweight but was alert and clinically stable. His vital signs were within normal limits for age. Physical examination revealed mild dehydration and conjunctival pallor. Abdominal examination demonstrated mild distension and localized tenderness in the right lower quadrant, without guarding, rebound tenderness, or palpable organomegaly. No abdominal mass was clearly appreciated on initial examination. Bowel sounds were present. Examination of other systems was unremarkable, with no peripheral lymphadenopathy, hepatosplenomegaly, or skin rash. Key laboratory findings on admission are summarized in Table 1.

**Table 1:** Key laboratory findings on admission

Laboratory test	Value	Normal range (nine years)
Hemoglobin	9.0 g/dL	11.5-15.5 g/dL
Ferritin	<6 ng/mL	20-100 ng/mL
White blood cell count	28.4 × 10⁹/L	4.5-13 × 10⁹/L
Erythrocyte sedimentation rate	56 mm/hr	<20 mm/hr
Lactate dehydrogenase	460 U/L	140-280 U/L
C-reactive protein	2.9 mg/L	<5 mg/L
Albumin	41 g/L	35-50 g/L

One month prior to the current admission, in August 2025, the patient underwent a surgical appendectomy for persistent abdominal pain. During surgery, an intraluminal, nonfixed, freely mobile cecal mass was incidentally identified. Its mobility led the surgical team to suspect a fecal mass rather than a neoplastic lesion. No intraoperative features suggestive of Crohn’s disease were observed, including terminal ileal thickening, creeping fat, serosal hyperemia, skip lesions, or mesenteric lymphadenopathy. Given the mass’s location near the ileocecal valve and terminal ileum, complete excision was not attempted to avoid extensive ileocecal resection and the associated risks of bowel injury, postoperative strictures, and significant morbidity in a child. The primary surgical goal was to obtain diagnostic tissue while minimizing operative risk. The appendix was removed, and multiple biopsies were obtained from the cecal mass. Histopathological examination demonstrated minimal histiocytosis, with no evidence of lymphoma, while the appendix showed nonspecific inflammatory changes. In view of ongoing clinical suspicion for inflammatory bowel disease or infectious ileocecal pathology, tissue samples were also submitted for bacterial, mycobacterial (including tuberculosis), and fungal cultures; no pathogenic organisms were isolated.

**Figure 1:**
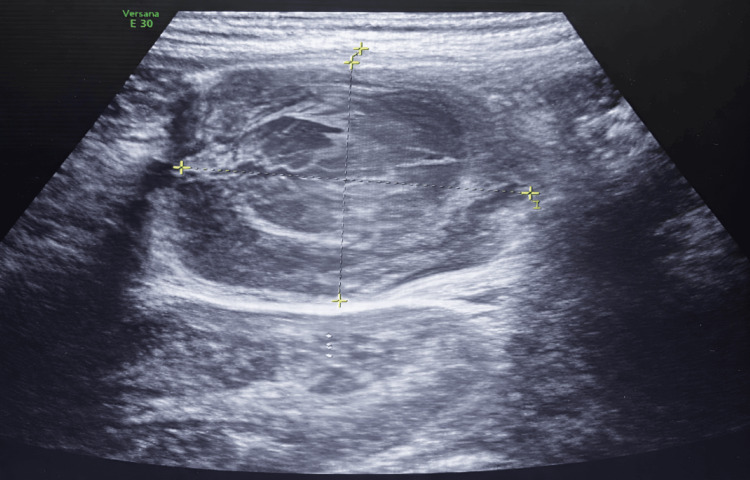
Abdominal ultrasound showing a right ileocecal mass with associated inflammatory changes

Ultrasound examination of the right lower quadrant revealed a 5 × 3.5 cm complex cystic mass containing turbid fluid in the right iliac region, closely associated with regional lymph nodes and adjacent thickened bowel loops. These findings were interpreted as consistent with complicated appendicitis or an appendiceal abscess (Figure 1).

**Figure 2:**
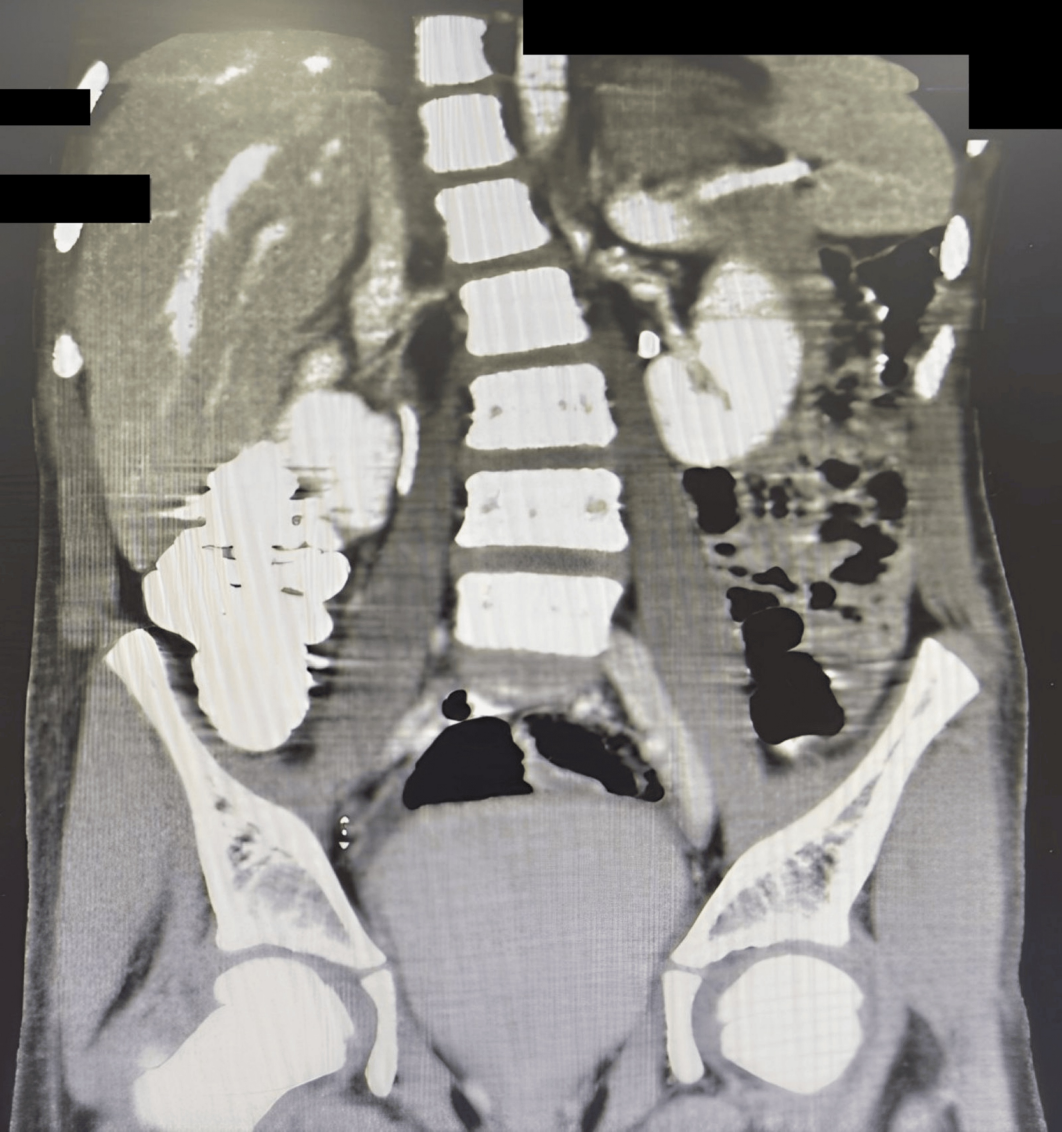
Coronal CT image showing an ill-defined fluid collection in the right iliac fossa, located between the terminal ileum and cecum, with mild mural thickening and faint peripheral enhancement

Subsequent CT enterography of the abdomen and pelvis demonstrated an ill-defined 3.7 × 3.6 × 3.0 cm fluid collection in the right iliac fossa with faint peripheral enhancement and mild terminal ileal wall thickening, favoring a peri-appendiceal inflammatory process or a possible infected enteric duplication cyst (Figure 2).

Despite an appendectomy, the patient’s symptoms persisted without improvement, including ongoing abdominal pain, diarrhea, and worsening anemia. Given the lack of clinical response, he was referred to the pediatric gastroenterology service for further evaluation, prompting admission for a comprehensive endoscopic assessment.

**Figure 3:**
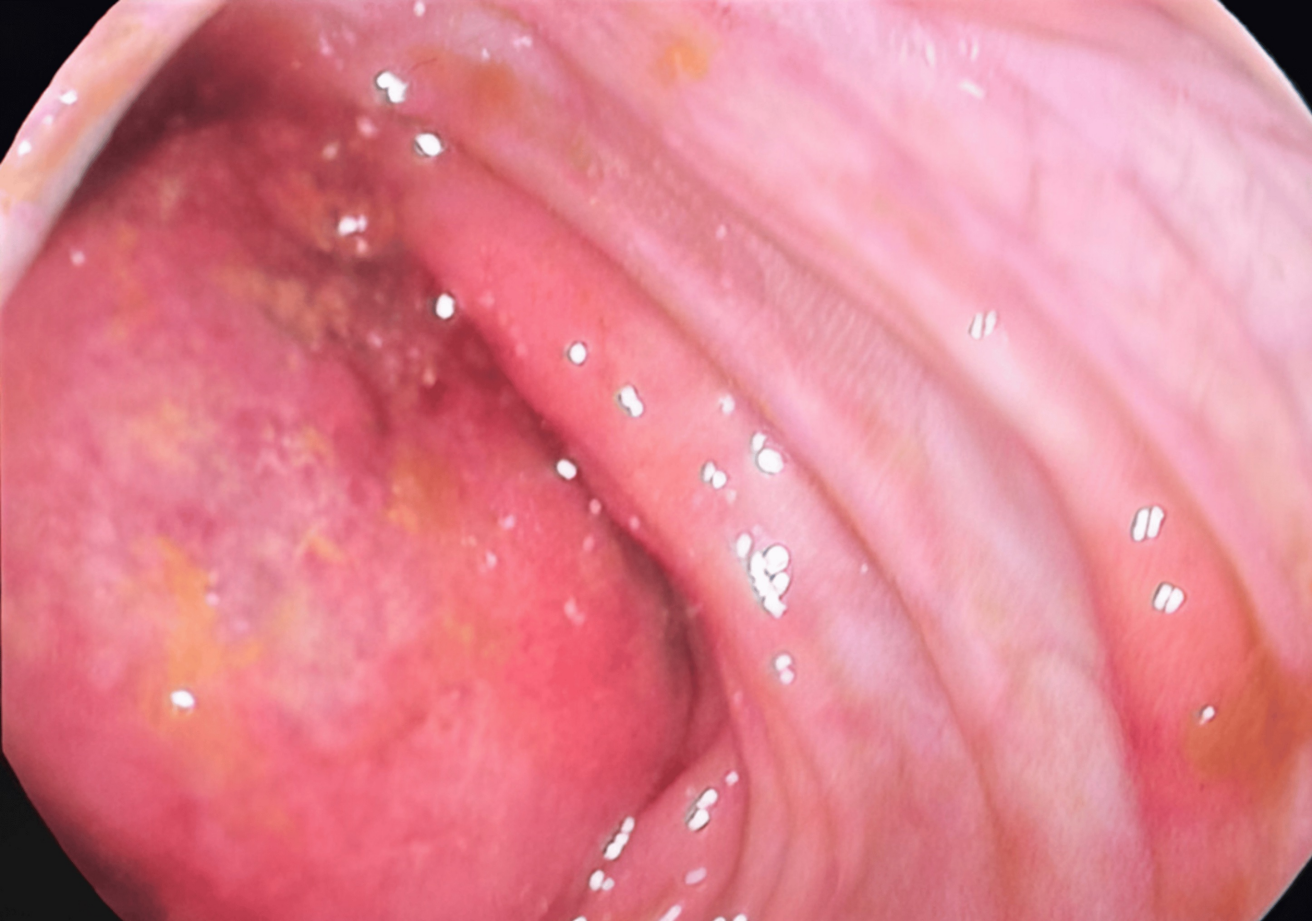
Endoscopic image showing a large, ulcerated cecal mass with extensive surface ulceration, inflammation, and exudate

Upper GI endoscopy revealed normal mucosa throughout the esophagus, stomach, and duodenum. Colonoscopy demonstrated a large obstructing intraluminal mass in the ascending colon, measuring approximately 10-15 cm in length. The colonoscope could not be advanced beyond the lesion. The mass appeared ulcerated and malignant in appearance (Figure 3).

**Figure 4:**
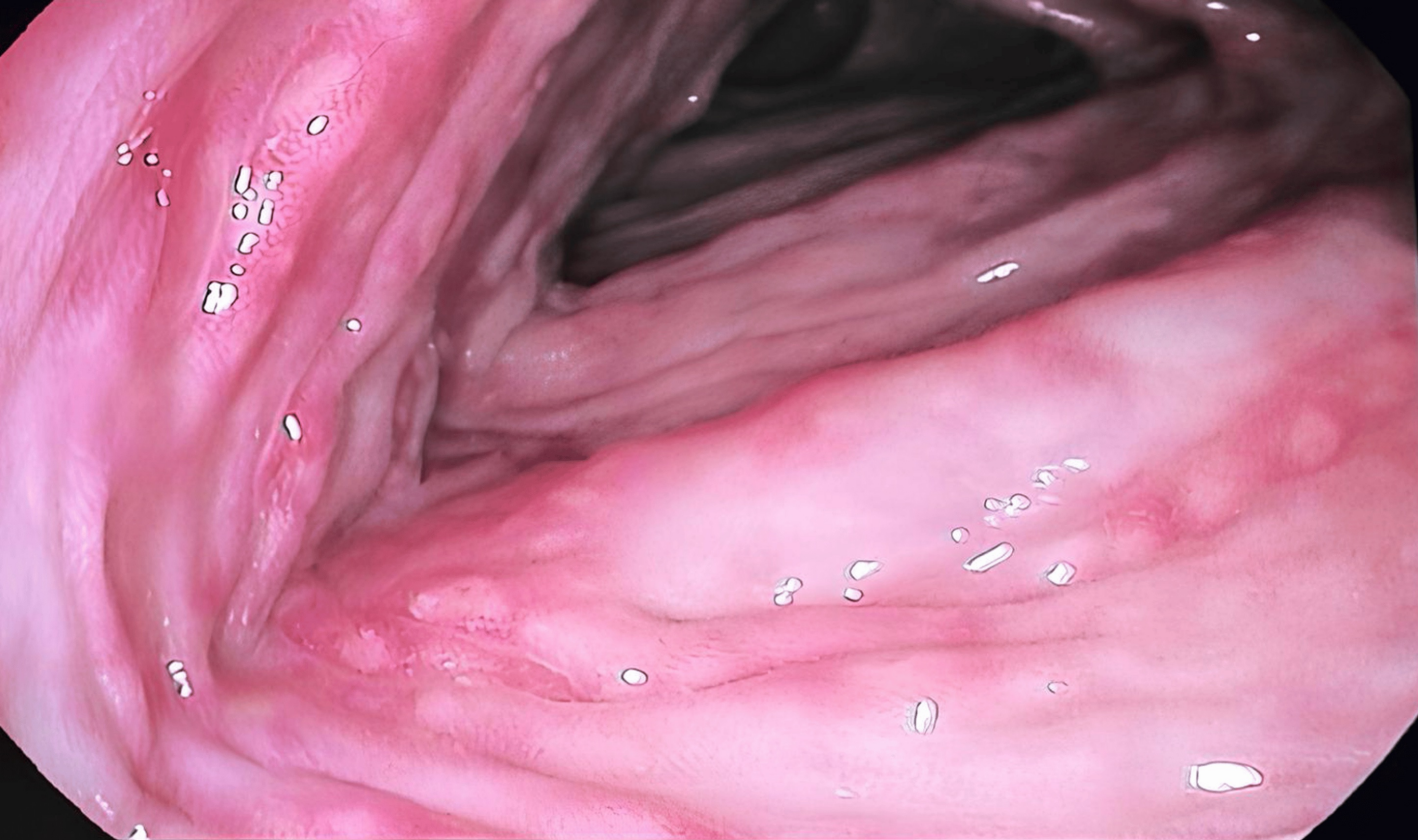
Multiple aphthous ulcerations with surrounding mild erythema and edema in the transverse colon

In addition, multiple aphthous ulcerations with surrounding mild erythema and edema were noted in the descending colon (Figure 4).

Multiple biopsies were obtained from the mass and the ulcerated colonic mucosa.

**Figure 5:**
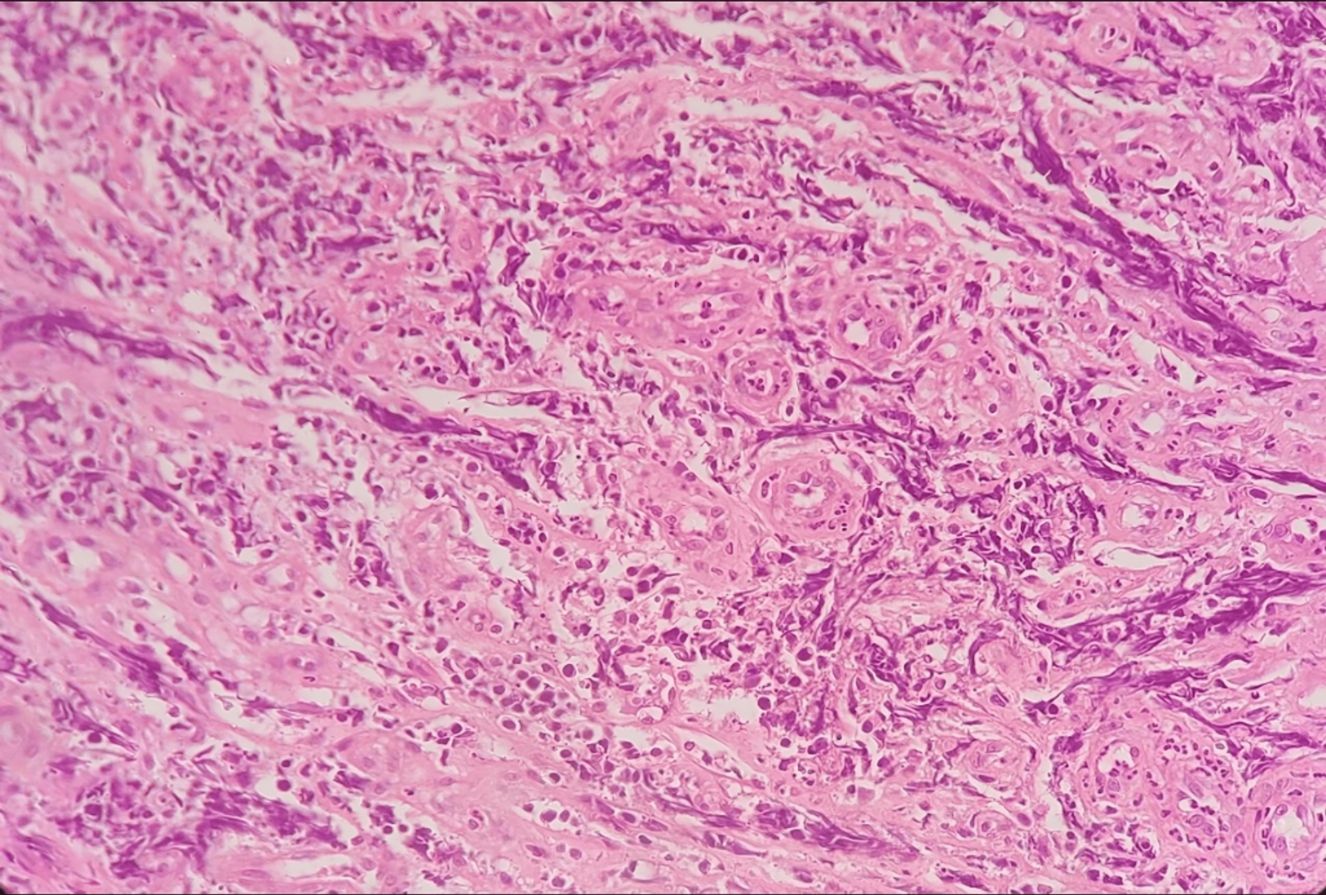
Histologic section showing sheets of monotonous small lymphocytes with prominent crush artifact No colonic glands are identified.

Histopathological examination of the biopsy specimens confirmed the diagnosis of BL, classified as high-grade B-cell lymphoma (Figure 5).

Immunohistochemical analysis demonstrated a Ki-67 proliferation index approaching 100%, indicating a highly aggressive tumor. Fluorescence in situ hybridization analysis provided definitive molecular confirmation, revealing rearrangement of the C-MYC gene in 78% of evaluated nuclei. A rearrangement of the BCL6 gene was also identified in 42% of nuclei, while the BCL2 gene showed a normal configuration.

Following diagnosis, a comprehensive staging workup was performed. Bone marrow aspirate and trephine biopsy, flow cytometry, and cerebrospinal fluid analysis were all negative for lymphomatous involvement. A post-diagnostic CT scan of the abdomen demonstrated a large ileocecal mass causing ileocecal intussusception, without evidence of bowel perforation or distant metastatic disease. The disease was classified according to the ANHL-1131 Group B protocol.

The patient was initiated on the RCOPADM1 Group B chemotherapy regimen. After eight weeks of chemotherapy, he demonstrated an excellent early therapeutic response, with post-COP imaging revealing a 36% reduction in tumor volume. Clinically, he showed marked improvement in abdominal symptoms, appetite, and weight trajectory, consistent with a favorable treatment response.

## Discussion

This case report describes a challenging diagnostic course in a pediatric patient with ileocecal BL and highlights several important clinical lessons. The most distinctive feature of this case is the significant delay in diagnosis despite early surgical intervention and an initial biopsy, a scenario that is infrequently reported in the literature. This case demonstrates the diagnostic complexity of GI BL and the potential pitfalls that may arise even when invasive evaluation is performed.

Presentation of BL as an acute abdomen is well recognized but remains diagnostically challenging. In a recent review by Tan et al., the majority of pediatric BL cases presenting with acute abdominal pain were initially misdiagnosed as appendicitis, with appendectomy being the most common initial intervention [3]. While the present case follows this general pattern, it differs in a critical respect. Although a cecal mass was identified intraoperatively and biopsied, the initial histopathological examination was nondiagnostic. This false-negative result contributed to a one-month delay in establishing the definitive diagnosis, during which the patient’s symptoms persisted, and the tumor progressed. This finding emphasizes that a negative biopsy does not reliably exclude malignancy when clinical suspicion remains high and reinforces the importance of continued vigilance and repeat diagnostic evaluation in the setting of persistent or unexplained symptoms [11].

The clinical course of this patient was further complicated by evolving features that closely mimicked multiple GI conditions. The initial five-month history of chronic abdominal pain, diarrhea, and weight loss was strongly suggestive of IBD. The presence of aphthous ulcerations in the colon during endoscopy further supported this diagnostic consideration. As reported by Erkan et al., GI lymphoma may closely resemble Crohn’s disease both clinically and radiologically, making differentiation particularly difficult in pediatric patients [8]. The subsequent acute exacerbation of pain leading to appendectomy, during which the mass was misinterpreted as a fecalith, added another layer of diagnostic complexity. This sequence of IBD mimicry, presumed appendicitis or fecal impaction, and postoperative persistence of symptoms represents a rare but instructive diagnostic trajectory [12].

Imaging findings also contributed to the diagnostic challenge. Initial ultrasound and CT findings were interpreted as consistent with benign inflammatory or infectious processes. Retrospectively, these findings likely represented early manifestations of the underlying malignancy. The documented increase in tumor size from approximately 3.7-5 cm on initial imaging to 10-15 cm at the time of endoscopy one month later clearly illustrates the rapid growth kinetics characteristic of BL and highlights the clinical consequences of diagnostic delay.

Laboratory abnormalities provided additional clues to the underlying diagnosis. The patient demonstrated marked leukocytosis and a significantly elevated erythrocyte sedimentation rate. Notably, lactate dehydrogenase levels were markedly increased, a well-established feature of high-grade lymphomas such as BL [13]. Elevated lactate dehydrogenase reflects high tumor turnover and tumor burden and has been consistently associated with disease activity and prognosis in BL [14-16]. In this context, the laboratory profile should have raised early concern for an underlying malignant process.

Despite the prolonged symptom duration and diagnostic delay following the initial surgical intervention, the patient demonstrated an excellent early response to chemotherapy. This outcome highlights the remarkable chemosensitivity of BL and provides an encouraging reminder that favorable outcomes can still be achieved even when diagnosis is delayed. Early recognition, prompt initiation of therapy, and adherence to established treatment protocols remain critical to optimizing outcomes in pediatric BL.

## Conclusions

This case of pediatric ileocecal BL illustrates a complex and protracted diagnostic course, marked by clinical features that closely mimicked several common pediatric GI conditions and by a significant delay in diagnosis despite prior surgical intervention and tissue sampling. The initial nondiagnostic biopsy represents a critical diagnostic pitfall in the evaluation of this highly aggressive malignancy. This report emphasizes the necessity of maintaining a high index of suspicion for underlying malignancy in children with persistent, progressive, or unexplained abdominal symptoms. It also reinforces that a negative initial biopsy does not exclude lymphoma when clinical and laboratory findings remain discordant, and that repeat evaluation with adequate and targeted tissue sampling is often essential to establish an accurate diagnosis.
